# Understanding West Nile virus ecology in Europe: *Culex pipiens* host feeding preference in a hotspot of virus emergence

**DOI:** 10.1186/s13071-015-0831-4

**Published:** 2015-04-09

**Authors:** Annapaola Rizzoli, Luca Bolzoni, Elizabeth A Chadwick, Gioia Capelli, Fabrizio Montarsi, Michela Grisenti, Josue Martínez de la Puente, Joaquin Muñoz, Jordi Figuerola, Ramon Soriguer, Gianfranco Anfora, Marco Di Luca, Roberto Rosà

**Affiliations:** Department of Biodiversity and Molecular Ecology, Research and Innovation Centre, Fondazione Edmund Mach, Via E. Mach. 1, 38010 San Michele all’Adige, Trento, Italy; Direzione Sanitaria – Servizio di Analisi del Rischio, Istituto Zooprofilattico Sperimentale della Lombardia e dell’Emilia Romagna, Via dei Mercati 13, 43100 Parma, Italy; Cardiff University, School of Biosciences, Biomedical Science Building, Museum Avenue, Cardiff, CF10 3AX, United Kingdom; Laboratory of Parasitology - Istituto Zooprofilattico Sperimentale delle Venezie, Viale dell’Università 10, 35020 Legnaro (Padova), Italy; Department of Veterinary Sciences, University of Torino, Largo Paolo Braccini 2, 10095 Grugliasco Torino, Italy; Department of Wetland Ecology Estación Biológica Doñana, Consejo Superior de Investigaciones Cientificas, Avda. Americo Vespucio s/n, 41092 Sevilla, Spain; Department of Sustainable Ecosystems and Bioresources, Research and Innovation Centre, Fondazione Edmund Mach, Via E. Mach. 1, 38010 San Michele all’Adige, Trento, Italy; Department of Infectious, Parasitic and Immune-Mediated Diseases, Istituto Superiore di Sanità, Viale Regina Elena, 299, 00161 Rome, Italy

**Keywords:** *Culex pipiens*, Feeding preference, Mosquito host selection, Blood meal analysis, Multinomial simulations, Behavioural bioassay, Reservoir host, *Turdus merula*, *Pica pica*

## Abstract

**Background:**

Understanding wildlife disease ecology is becoming an urgent need due to the continuous emergence and spread of several wildlife zoonotic diseases. West Nile Virus (WNV) is the most widespread arthropod-borne virus in the world, and in recent decades there has been an increase both in geographic range, and in the frequency of symptomatic infections in humans and wildlife. The principal vector for WNV in Europe is the common house *Culex pipiens* mosquito, which feeds on a wide variety of vertebrate host species. Variation in mosquito feeding preference has been described as one of the most influential parameters driving intensity and timing of WNV infection in the United States, but feeding preferences for this species have been little studied in Europe.

**Methods:**

Here, we estimated feeding preference for wild *Cx. pipiens* in northern Italy, using molecular analysis to identify the origin of blood meals, and avian census to control host abundance variations. Additionally, we used host bird odour extracts to test experimentally mosquito preferences in the absence of environmental variations.

**Results:**

For the first time, we demonstrate a clear feeding preference for the common blackbird (*Turdus merula*), both for wild collected specimens and in the lab, suggesting a potential important role for this species in the WNV epidemiology in Europe. A seasonal decrease in abundance of blackbirds is associated with increased feeding on Eurasian magpies (*Pica pica*), and this may be linked to seasonal emergence of WNV in humans. Feeding preferences on blackbirds are more marked in rural areas, while preference for magpies is higher in peridomestic areas. Other species, such as the house sparrow (*Passer domesticus*) appear to be selected by mosquitoes opportunistically in relation to its abundance.

**Conclusions:**

Our findings provide new insights into the ecology of *Cx. pipiens* in Europe and may give useful indications in terms of implementing targeted WNV surveillance plans. However, a clearer understanding of spatio-temporal variations of *Cx. pipiens* feeding preferences, and targeted studies on reservoir competence for WNV for these species are therefore now urgently needed as this is essential to describe disease dynamics and quantify virus transmission risk.

## Background

Zoonoses are infections that can be transmitted from vertebrate animals to humans. Approximately 60% of emerging infectious diseases in humans, and almost all recent pandemic threats, have had a zoonotic origin [1]. The huge economic and social burden of zoonotic disease drives a pressing need to better understand wildlife disease ecology, in order to describe disease dynamics and quantify hazard, thereby enabling targeted surveillance and providing support for sustainable disease control.

In the case of vector-borne zoonoses, such as arboviruses (arthropod-borne viruses), spill-over events are the result of complex ecological interactions affecting pathogens, vectors, and their hosts [2]. Transmission intensity is determined by both ‘reservoir competence’, defined as the relative ability of a reservoir host species to maintain and transmit the pathogen to a competent vector, and contact rates between hosts and vectors [3]. Variables such as climate, habitat structure, and the relative abundance and behaviour of vectors and hosts all contribute to the complexity that characterises the dynamics of transmission of vector-borne pathogens [4-7].

Mosquitoes are among the most important vectors, and transmit some of the most threatening infectious diseases in the world, such as malaria, dengue fever, Rift Valley fever, and West Nile virus (WNV) [8,9]. Current understanding of mosquito-borne pathogen transmission is underpinned by the simple theoretical framework developed by Ross [10] and Macdonald [11], providing testable predictions on which control decisions can be based [12,13]. A central assumption of most models founded on this framework is that transmission occurs homogenously in well mixed populations, an assumption that has, however, been called into question at a range of spatial scales [13].

Species-specific variation in both contact rates and infectiousness drives considerable heterogeneity in pathogen transmission [14]. Some mosquito species are generalist and express opportunistic feeding behaviour, while others are specialists and feed preferentially on selected hosts [15,16]. Host feeding preferences vary among mosquito species and populations, and are affected by factors including season, mosquito nutritional status, host behaviour or mosquito learning over time [17-22]. Studies of mosquito feeding preference are essential to understand the ecology of arbovirus transmission. In fact, at a population level, such feeding preferences may enhance or reduce transmission if vectors feed on competent or incompetent hosts, respectively [23]. In order to distinguish between opportunistic and specialized feeding behaviours, blood meal analysis alone is insufficient, as it fails to take into account differences in host availability and behaviour [19]. Recognising this, Hassan *et al.* [24] proposed a ‘feeding preference index’, which examines the number of blood meals from a given host species as a fraction of blood meals from all identified hosts, and compares them with the proportional abundance of that species in the host community. By combining this information with choice experiments in the laboratory, it is possible to test mosquito preferences in the absence of confounding factors [25].

West Nile Virus is a multi-host pathogen of the genus *Flavivirus* belonging to the Japanese encephalitis sero-complex. Reported for the first time in Uganda in 1937 [26], it is now considered the most widespread arbovirus in the world [2]. Maintained in a bird-mosquito transmission cycle, WNV can affect a wide range of vertebrates including humans and horses, the last two acting epidemiologically as ‘dead-end’ hosts that are susceptible to infection but do not transmit the virus [17]. Recent analyses of vector feeding preferences in the New World have greatly enhanced the understanding of WNV transmission dynamics (e.g. [17,24]). Models suggest that feeding preference is among the most influential parameters driving intensity and timing of peak WNV infection in mosquito vectors, and is essential for modelling transmission dynamics and predicting outbreaks [3]. Combining analysis of host preference, abundance, host behaviour and reservoir competence, Kilpatrick *et al.* elegantly demonstrated that the American robin (*Turdus migratorius*) acted as an unexpected ‘super-spreader’ of the WNV in North America [14,22].

Despite a much longer history of virus circulation in the Old World [27], a detailed understanding of virus ecology and vector-host interactions is still lacking in Europe. Currently, a number of field studies have identified mosquito hosts using blood-meal analysis (e.g. in Czech Republic [28]; Spain [29]; Italy [30]; Portugal [31]; and Israel [32]). However, to our knowledge, there has been no assessment of host preference in Europe either by integrating blood meal analyses with host availability in the field, or by choice experiments in the laboratory.

The current study aimed to quantify feeding preferences of the common house mosquito *Cx. pipiens*, considered the principal vector of West Nile virus in Europe [33], within a hot spot of WNV circulation in northern Italy [34,35]. Using two complementary approaches we first identified the feeding preference of *Cx. pipiens* in nature by combining analysis of blood meal origin with assessment of host availability, and we analysed seasonal and spatial variation in host preference. Second, we analysed intrinsic preferences in the absence of confounding variables (environmental variations, host abundance and behaviour) by testing the relative attractiveness of odour extracts from wild birds for a laboratory colony of *Cx. pipiens*. Together, these data help to elucidate the relative importance of specific bird species to the epidemiology of WNV in Europe.

## Methods

### Mosquito feeding preference indices in the field

#### Mosquito collection

**Figure 1:**
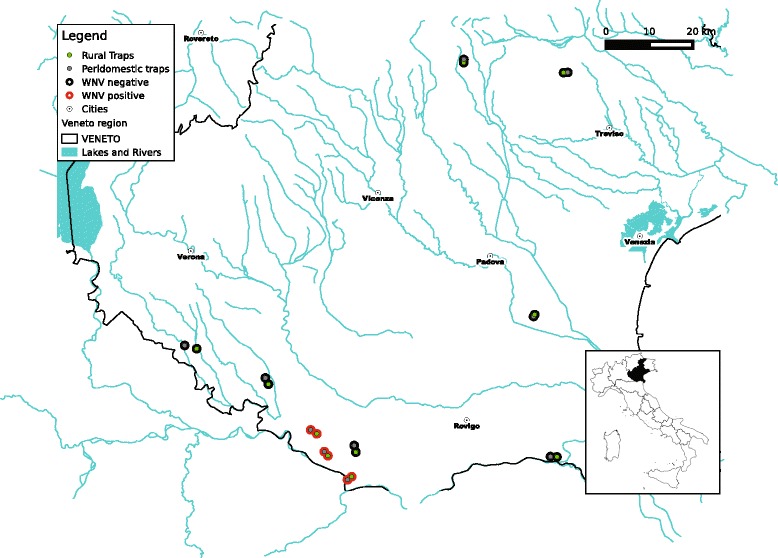
**Map of the study area.** Mosquito trapping sites in Veneto region, Italy. The lower boundary corresponds to the Po Valley (Pianura Padana). Inset indicates the location of the Veneto Region. Positivity for WNV in mosquitoes was recorded in the period 2010-2012.

Mosquitoes were collected in Veneto region (north-eastern Italy), where WNV has been detected in the mosquito species *Cx. pipiens*, in animals and humans since 2008 [35-37]. Mosquito traps were located in ten localities within Veneto, a region characterized by mild climate, intensive agriculture and animal husbandry, medium-small urban settlements, irrigated areas, wetlands and marshes, with abundant mosquito and bird populations. Ten mosquito sampling localities were selected where one trap was positioned in a rural environment and the other one in a peridomestic environment (Figure 1). Data on WNV occurrence in mosquitoes at each locality was obtained from a regional surveillance program [38] with three of the sampling localities (6 traps) recorded as WNV positive and the other seven (14 traps) as WNV negative (Figure 1). Mosquitoes were collected from May to October 2012, using BG-sentinel traps baited with BG-lure attractant (Biogents AG, Regensurg, Germany). Once a week, each trap was set in the morning and checked after 24 hours. Every two weeks the traps were additionally baited with dry ice as a source of carbon dioxide. Captured mosquitoes were stored at -80°C and identified to species using morphological keys for Italian Culicidae [39]. Blood fed females were stored individually at -80°C, in centrifuge tubes with 1 ml of ethyl alcohol 70% until analysis.

#### DNA extraction and identification of mosquito blood meal

The abdomen of each mosquito female with a recent blood meal was separated from the head-thorax in a sterile Petri dish, using sterile tips. The DNA contained in each abdomen was isolated using the DNeasy Blood and Tissue® kit (QIAGEN, Hilden, Germany) following company specifications (see [40]). We used a nested- PCR protocol that selectively amplifies 758 bp of the vertebrate mitochondrial Cytochrome c Oxidase Subunit I (COI) gene to identify blood meal origin [41]. Negative controls (solutions prepared in an identical manner, with no mosquito extract) were included in each PCR reaction plate. After sequencing of the amplified COI fragment, we used the identification engine implemented in the Barcode of Life Data (BOLD) Systems database (http://www.barcodinglife.org/) to assign COI sequences to particular species.

#### Census of wild birds and mammals

To quantify availability of vertebrate hosts in relation to mosquito feeding preferences, we carried out a survey once a month, within 5 days from each trapping period.

Bird counts were obtained using both sightings and calls. Counts started at sunrise, and carried out for 6 minutes at each of twenty locations, these being at the mosquito trap site and at 4 points 200 meters from the trap site in each cardinal direction. For each observation (visual and auditory) species and number of individuals were recorded. Where additional species were observed outside count periods, records were added to species lists.

#### Calculation of feeding preferences

Data on avian host abundance and mosquito feeding were used to compute feeding preference indices (*P*_*i*_) of *Cx. pipiens* mosquitoes, defined as:

1 $$ {P}_i=\frac{f_i}{a_i} $$

where *f*_*i*_ represents the fraction of total blood meals taken by *Cx. pipiens* from host *i* and *a*_*i*_ represents the density of species *i* divided by the total density of the avian community [24]. Where *P*_*i*_ = 1, the fraction of blood meals from species *i* is directly proportional to host species abundance, and can therefore be said to represent “opportunistic” feeding habits. Where *P*_*i*_ < 1, the species is under-represented in blood meals in respect to host abundance, and is therefore considered “avoided”. Conversely, where *P*_*i*_ > 1, the species is considered “preferred”. Several species present in the avian host community were not detected in blood meal samples. For those species it was necessary to determine whether this absence was due to avoidance, or to insufficient sample size. For those species, we assigned a value *f*_0_ = (1-0.5^1/*n*^) which represents half the probability of not observing any blood meals from this species given the total blood meal sample size, *n*. Then, for species that were not detected in mosquito blood meals, we assumed a conservative estimate *P*_*i*_ = *f*_0_/*a*_*i*_ if the species was significantly avoided or *P*_*i*_ = 1 if not. To predict the distribution of expected blood meals based on a null hypothesis of opportunistic feeding, we performed 10,000 multinomial simulations based on census data (after [14]). Following Hassan *et al.* approach [24], we estimated for each host species the probability of observing a larger [or smaller] than unity feeding preference index by computing the fraction of the 10,000 simulations in which *P*_*i*_ was higher [or lower] than 1.

In order to compare the pattern of mosquitoes feeding habits in peridomestic and rural sites, we computed two different feeding preference indices as in (1) using blood meals and avian census data obtained with traps in peridomestic, *P*_*i.peridomestic*_, and rural, *P*_*i.rural*_, localities, respectively. To test the significance of these differences, we used multinomial simulations where samples of blood meals and host species abundances in each simulation were extractions from multinomial distributions with probabilities *f*_*i.peridomestic*_ and *a*_*i.peridomestic*_ in peridomestic sites and *f*_*i.rural*_ and *a*_*i.rural*_ in rural sites, respectively. (Where *f*_*i.peridomestic*_ [*f*_*i.rural*_] represents the fraction of total blood meals taken by *Cx. pipien*s from host *i* in peridomestic [rural] sites and *a*_*i.peridomestic*_ [*a*_*i.rural*_] represents the density of species *i* over the total density of the avian community estimated in peridomestic [rural] sites.) For each host species we estimated the probability of observing a larger [or smaller] feeding preference index in peridomestic than in rural sites by computing the fraction of the 10,000 simulations where the difference in feeding preferences indices, *P*_*i.peridomestic*_ - *P*_*i.rural*_, was positive [or negative].

Similarly, in order to investigate the seasonal patterns of mosquito feeding habits, we computed two different feeding preference indexes as in (1) by using blood meals and avian census data obtained in the early (May-June, *P*_*i.early*_) and the late (July-September, *P*_*i.late*_) mosquito activity season. These two periods were selected in order to test whether mosquito feeding habits are affected by the seasonal changes in the behaviour of some avian species. For instance, frugivorous birds, such as the common blackbird (*Turdus merula*, hereafter, blackbird), at the end of its breeding season (in July), moves from nesting areas to sites rich in fruit bearing plants [42,43]. Other species, such as barn swallow (*Hirundo rustica*), after the breeding season move for gregarious foraging or start migrating [44]. These behavioural changes modify the composition of the avian host community and are therefore likely to affect the feeding patterns of *Cx. pipiens*.

Finally, we used the same method to compare feeding preference indices between sites where WNV occurrence in mosquitoes has, or has not, been observed (*P*_*i.WNV+*_, or *P*_*i.WNV-*_, respectively) during 2010-2012 [38]. Simulations were performed using MATLAB 7.10.0 (The Mathworks, Inc.).

### Mosquito feeding preferences in the laboratory

#### Collection of test subjects

Four wild bird species were selected based on the outcome of field census and preference analyses: blackbird and magpie*,* both abundant and preferred in the field; house sparrow, abundant and fed on opportunistically, and Eurasian blackcap (*Sylvia atricapilla*)*,* neither abundant nor preferred but displaying feeding and breeding habits similar to blackbird. All test subjects were captured using mist-nets during spring and summer 2013. Captures were carried out by an ornithologist authorized by the National Institute for Environmental Protection and Research ISPRA and the research protocol was approved by Local Wildlife Management and Veterinary Welfare Committees. Since both sex and age might influence the composition of odour bouquet emitted by birds [45-47], only adult males were considered in the experiments. Four males of each species were captured.

Mosquitoes used in the experiments derived from eggs laid by gravid insects captured in the field and reared in the laboratory at the National Institute of Health in Rome. Only adult female mosquitoes were employed in behavioural bioassays.

#### Collection of odour extracts

Differences in odour composition have previously been shown to be significant in determining host preference in mosquitoes [25], and odour extracts have been used to test mosquito host preference [45-47]. Odour extracts were used rather than live birds in the current study in order to minimise animal welfare issues.

Once in the laboratory, each bird was placed in an airtight polypropylene desiccator (Carlo Erba Reagents S.p.A., Milan, Italy) of 240 mm (for blackbirds and magpies) or 140 mm diameter (house sparrows and blackcaps), according to the size of the bird. Charcoal-filtered air was pumped through the system at 150 ml/min and over a Porapak Q cartridge that contained 50 mg of adsorbent, for one hour for each animal [48]. The birds were then immediately released at the site of capture. Volatiles were desorbed by eluting the cartridge with 600 *μ*l of redistilled hexane. The extracts were stored at -20°C until used. To avoid cross contamination, the polypropylene desiccator was cleaned with denatured alcohol between each use.

#### Behavioural trials

All trials were conducted in August and September 2013 during the physiological peak of host-seeking activity of *Cx. pipiens* (about 2 hours after sunset) [49] in a room with infrared light to mimic the physiological crepuscular-nocturnal activity of this species [50,51]. A plastic Petri-dish (diameter 25 cm, height 4 cm) was used as the test arena, and was placed centrally in a white, uniformly illuminated box (50 cm x 30 cm, 100 lux), to prevent distraction by surrounding objects.

The bottom of the dish was covered with a filter paper disk. Two additional pieces of filter paper (1 cm^2^) were placed on top of this paper, on opposite sides of the Petri dish: one was soaked with 40 μl of odour extract from the selected bird species, while the other was soaked with 40 μl of hexane, thus acting as control. The test arena was demarcated into three equally sized sectors: one lateral sector including the odour extract, a 5 cm wide central strip, and one lateral sector including the control. For each bird species, the test was repeated using extracts from four different adult male individuals.

Using a manual aspirator, insects were taken from their cages, inserted individually through a central hole in the lid of the Petri dish and observed for 7 min. The number of observed mosquitoes varied among tests ranging from 60 to 80 individuals (specifically: 60 blackbirds, 60 magpies, 60 house sparrows, and 80 Eurasian blackcaps). The time spent in each of the sectors was recorded. Insects that settled for at least 70% of the test duration in one of the lateral sectors were scored as having a preference. Individuals that spent less than 70% of the time in either lateral sector, or remained in the central sector, were counted as exhibiting no preference.

For each test, the positions of the disks of filter paper were randomly rotated to avoid any positional effect. In addition, preliminary analyses were carried out to test for positional bias, by conducting trials first with both filter papers soaked with hexane, and then both with odour extract (in this case, from *T. merula*), in each case with 30 mosquitoes. For all the tests individual insects were used only once to avoid bias from previous exposure.

#### Calculation of odour preference

For each host species, the number of individuals that chose the sector with the odour source was compared with those choosing the control (hexane). Individuals exhibiting no preference were excluded. A chi-square test was used to compare the number of mosquitoes that chose the odour source versus those that chose the control sector within each test extract bioassay. Differences between bird extracts were evaluated by contingency table analysis based on chi-square followed by a Ryan’s multiple comparison test on proportions (p < 0.05) [52]. Both chi-square tests were Yates corrected.

## Results

### Bird census

**Figure 2:**
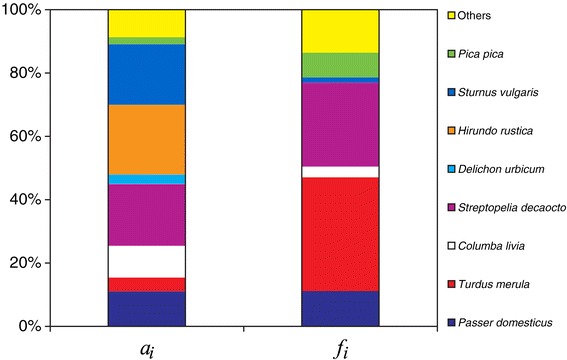
**Avian abundance and blood meal origins.** Relative abundance of birds (*a*_*i*_) and percentage of *Cx. pipiens* blood meals from bird species (*f*_*i*_) at site traps in Veneto.

Censuses showed a total of 31 wild avian species, including over two thousand individuals. Eight species dominated the bird community, representing more than 90% of the total number of individuals (Figure 2). They were (from the most to the least abundant) barn swallow, Eurasian collared dove (*Steptopelia decaocto*), common starling (*Sturnus vulgaris*), house sparrow, rock dove (*Columba livia*), blackbird, common house martin (*Delichon urbicum*) and magpie.

### Mosquito collection and feeding preference indices in the field

We collected 259 blood-fed females in total, identified as *Cx. pipiens* (206), *Anopheles maculipennis comple*x (39), *Aedes albopictu*s (12) and *Ochlerotatus caspiu*s (2). We computed feeding preference indices only for *Cx. pipiens* as sample sizes for other mosquito species were insufficient.

A total of 188 hosts of 31 different species were identified from *Cx. pipiens* blood meals. Of these, 144 (77%) were avian of which 117 (62%) were wild birds and 27 (14%) domestic. The remaining 43 (22.9%) were mammals, of which 13 (6.9%) were humans, and one (0.5%) reptile.

Four species (blackbird, Eurasian collared dove, house sparrow, and magpie) were the origin of 81% (95/117) of blood meals coming from wild avian species (Figure 2). The other 22 blood meals came from 14 wild bird species. Analyses of feeding preference indices of the eight most abundant bird species, derived from *n* = 117 blood meal samples, indicate that blackbird and magpie were significantly preferred by *Cx. pipiens* while collared dove was marginally preferred (*P*_*blackbird*_ = 8.25, p < 0.001; *P*_*magpie*_ = 3.54, p < 0.001; *P*_*collared_dove*_ = 1.36, p = 0.056). Rock dove and common starling were significantly avoided (*P*_*rock_dove*_ = 0.34, p < 0.01; *P*_*starling*_ = 0.089, p < 0.001). Despite their high abundance, neither common house martin nor barn swallow were detected in blood meals, suggesting that these species were significantly avoided (*P*_*house_martin*_ = 0.14, p < 0.05; *P*_*barn_swallow*_ = 0.019, p < 0.001). Finally, *Cx. pipiens* fed on house sparrow in proportion to its abundance (*P*_*house_sparrow*_ = 1.01, p > 0.05). Sample size constraints prevented calculation of feeding preference indices for the other less abundant wild bird species. Domestic species were excluded as census data were unrepresentative of abundance; also, despite their relatively high occurrence in blood meals (e.g. domestic chicken were identified in 21 cases, 14.5% of avian species) their role in circulation of WNV is unimportant as they are not deemed competent hosts.

**Figure 3:**
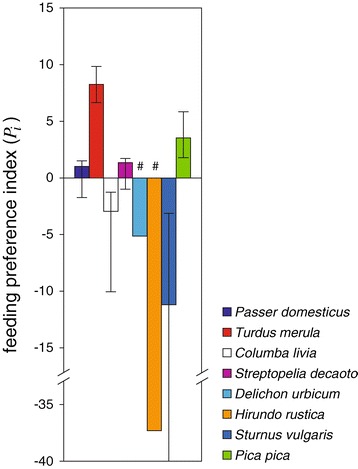
***Culex pipiens*** **feeding preferences for most abundant bird species.** Feeding preference indexes (*P*_*i*_) of *Cx. pipiens* mosquitoes and 95% confidence interval for the eight most abundant bird species in the Veneto region. Positive values are preferences; negative values designate avoidance and are calculated as (-1/*P*_*i*_). Species marked ‘#’ are calculated as conservative estimates (see text).

For the four non-avoided species for which a sufficiently large sample size was available (see Figures 2 and 3), we were able to compute feeding preferences distinctly in peridomestic vs. rural areas, in different seasons, or in areas with or without recorded WNV circulation.

**Figure 4:**
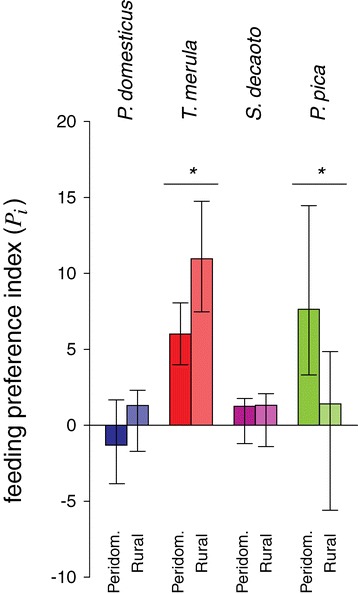
**Spatial** **variation of mosquito feeding preferences between peridomestic and rural sites.** Feeding preference indexes (*P*_*i*_) of *Cx. pipiens* mosquitoes of the most notable bird species in Veneto region in peridomestic and rural sites. Asterisks indicate statistical differences between areas (*: p < 0.05).

**Figure 5:**
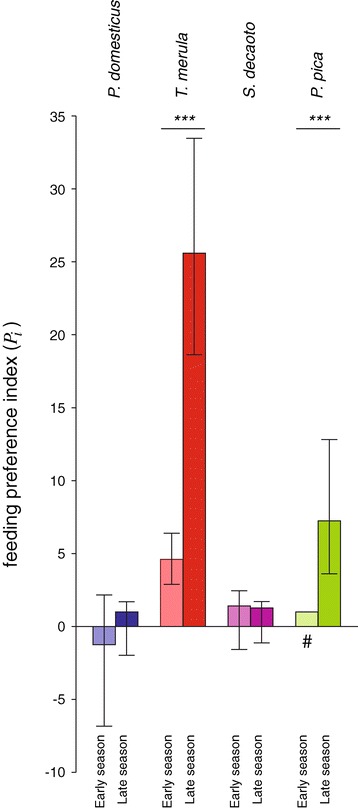
**Temporal variation of mosquito feeding preferences during mosquito activity season.** Feeding preference indices (*P*_*i*_) of *Cx. pipiens* mosquitoes of the most notable bird species in Veneto region in early season (May-June period) and late season (July-September period). Columns with hash key (#) are conservative estimates (see text). Asterisks indicate statistical differences between periods (***: p < 0.001).

Preference for blackbird was expressed more strongly in rural than in peridomestic areas while preference for magpie exhibited the opposite pattern; for collared dove and house sparrow, no significant differences were observed (Figure 4: *n*_*rural*_ = 53, *n*_*peridomestic*_ = 64, *P*_*blackbird.rural*_ = 10.97, *P*_*blackbird.peridomestic*_ = 6.01, p < 0.05; *P*_*magpie.rural*_ = 1.41, *P*_*magpie.peridomestic*_ = 7.65, p < 0.05). Preferences for blackbird and magpie were observed more strongly in the late than during the early part of the season, while preferences for collared dove and house sparrow were not significantly different between the two periods (Figure 5: *n*_*early*_ = 33, *n*_*late*_ = 84, *P*_*blackbird.late*_ = 25.58, *P*_*blackbird.early*_ = 4.60, p < 0.001; *P*_*magpie.late*_ = 7.25, *P*_*magpie.early*_ = 1, p < 0.001).

**Figure 6:**
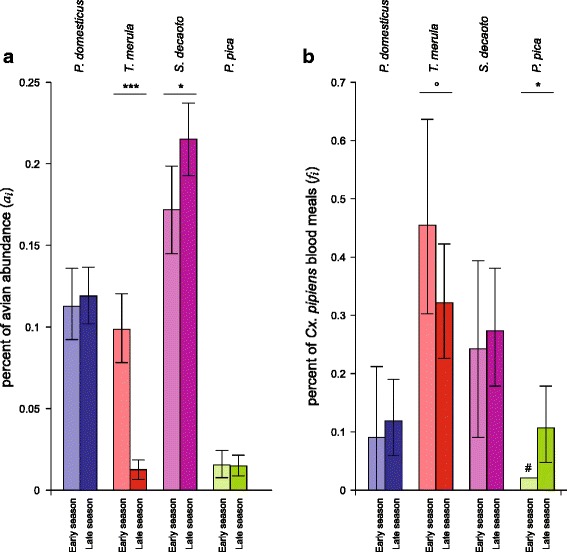
**Temporal variation of avian abundance and blood meal origins during mosquito activity season.** Percent of avian abundance (*a*_*i*_) **(panel a)** and percent of *Cx. pipiens* blood meals (*f*_*i*_) **(panel b)** for the most notable bird species in Veneto region. Early season: May-June period; late season: July-September period. Columns with hash key (#) are conservative estimates (see text). Asterisks indicate statistical differences between periods (°: p < 0.1, *: p < 0.05; ***: p < 0.001).

Figure 6 shows separately the seasonal change between the early and late periods for avian relative abundance (panel a) and for the proportion of blood meals (panel b). The increase in preference index for blackbird and magpie arose from differing causes: for blackbird, abundance was significantly less in the late season but was not accompanied by a decrease in the frequency of blood meals on this species; while for magpie, the abundance remained stable but the proportion of blood meals was greater in the late season.

During the latter part of the season, we also observed an increase in the number of *Cx. pipiens* bites on humans, from 2 bites (3.6% of the total blood meals) in the early season to 11 bites (8.3%) later in the season. However, sample size for bites on humans was too small, and this increase was not statistically significant.

**Figure 7:**
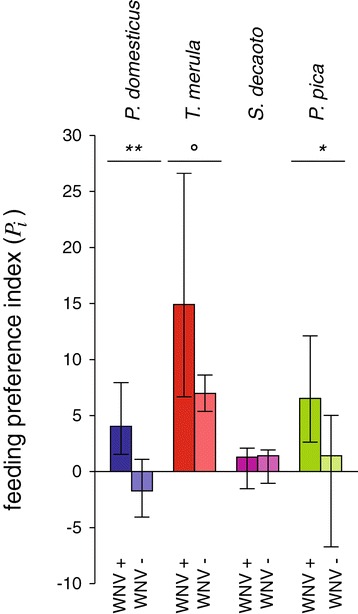
**Spatial** **variation of mosquito feeding preferences between WNV positive and WNV negative sites.** Differences in feeding preference indexes (*P*_*i*_) of *Cx. pipiens* mosquitoes of the notable non-avoided bird species in sites where West Nile virus (WNV) circulation in mosquitoes has been observed, WNV+, or not, WNV-, in Veneto region in the 2010-2012 time span. Asterisks indicate statistical differences between periods (°: p < 0.1; *: p < 0.05; **: p < 0.01).

A significant preference was observed for house sparrow within sites positive for WNV (WNV+) while no preference was detected for this species in areas negative for WNV circulation (WNV-) (Figure 7: *n*_*WNV+*_ = 39, *n*_*WNV-*_ = 78 *P*_*house_sparrow.WNV+*_ = 4.04, *P*_*house_sparrow.WNV-*_ = 0.58, p <0.01). Preference for magpie was significantly higher in WNV+ areas, while the preference for blackbird was marginally higher, and feeding preference for collared dove exhibited no significant difference between WNV+ and WNV- sites (*P*_*magpie.WNV+*_ = 6.52, *P*_*magpie.WNV-*_ = 1.41, p <0.01; *P*_*blackbird.WNV+*_ = 14.91, *P*_*blackbird.WNV-*_ = 6.97, p = 0.059).

### Mosquito feeding preferences in the laboratory

Odour extract solutions collected from all four bird species were attractive to *Cx. pipiens* in respect to the control (house sparrow: chi-square = 4.16, df = 1, p < 0.05; Eurasian blackcap: chi-square = 7.15, df = 1, p < 0.01; blackbird: chi-square = 28.88, df = 1, p < 0.001; magpie: chi-square = 4.33, df = 1, p < 0.05) (Table 1). Comparisons among species indicated that blackbird extracts were significantly more attractive than extracts from all other species (chi-square = 23.6, df = 3, p < 0.001; Ryan’s test, p < 0.05) (Table 1). Extracts from the other 3 species did not differ from each other in attractiveness (Ryan’s test, p < 0.05) (Table 1). The preliminary trial using paired hexane/paired odour extract excluded the possibility of positional bias (hexane: chi-square = 0.043, df = 1, p = 0.83; blackbird extract: chi-square = 0.07, df = 1, p = 0.79).

**Table 1:** **Olfactory responses of** ***Cx. pipiens*** **females to odour extracts of selected bird species**

**Bird species**	**N (%) odour**	**N (%) control**	***χ*** ^**2**^	**d.f.**	**p(** ***χ*** ^**2**^ **)**	**Ryan’s test**
Blackbird	39 (90.7)	4 (9.3)	28.88	1	<0.001	a
Eurasian blackcap	49 (66.2)	25 (33.8)	7.15	1	<0.01	b
Eurasian magpie	34 (65.4)	18 (34.6)	4.33	1	<0.05	b
House sparrow	54 (64.8)	35 (35.2)	4.16	1	<0.05	b

Columns description. N (%) odour: number and percentage of mosquitoes that exhibited preference to the odour extract solution; N(%) control: number and percentage of mosquitoes that exhibited preference to the solvent; *χ*2 statistics: chi-square test comparing the proportion of mosquitoes choosing odour vs. control for each bird species; Ryan’s test: comparison of proportions of mosquitoes choosing the odour coming from different bird species (rows with the same letter indicate that proportions are not statistically different at 0.05 level).

## Discussion

Our field study showed that bird species were not bitten by mosquitoes in proportion to their abundance. This supports the conclusion that the overall abundance of avian species is likely to be a poor indicator of importance in disease transmission, as has been demonstrated in the US [14,18,24]. We identified four bird species, the blackbird, the house sparrow, the magpie and the collared dove as the species most frequently bitten by *Cx. pipiens*. This result confirms previous studies on blood meal analysis conducted in European countries showing that *Cx. pipiens* fed most frequently on birds belonging to order Passeriformes [28]. Other European studies have reported both the house sparrow [29-31] and blackbird [30,31] as the most frequently occurring species in *Cx. pipiens* blood meals. Here, we found in addition that collared dove and magpie were abundant in mosquito blood meals, but that only blackbird and magpie were significantly preferred, while house sparrow and collared dove were fed upon opportunistically.

Previous studies have shown that the degree of blood meal digestion status of fed mosquitoes can alter the host composition identified in blood meal analysis [19,53]. However, in [19,53] mosquito sampling procedures were different in respect to our study; in particular, we used BG traps to collect engorged mosquitoes, while in [19,53] Thiemann *et al.* used CO_2_-CDC traps and gravid traps, as well as aspirating mosquitoes from resting sites. In a recent study [54] it has been observed that the number of freshly engorged mosquitoes collected with BG traps is higher than using CDC traps. Following these results, although in this study we did not collect quantitative data on the status of the blood meals, we can affirm that most of the blood meals identified at host species level derived from fresh fully engorged females. In fact, analysing fresh fully engorged females is essential to increase the success of host identification. Martínez-de la Puente *et al.* [40] showed that Sella score, a measure of the degree of blood meal digestion status, significantly affects the success of blood meal identification, with a significant drop in success of host identification for mosquitoes containing a blood meal in an advanced stage of digestion (Sella score higher than 5, see [40]).

The preference of *Cx. pipiens* for the blackbird was confirmed by the combination of two independent methods: the molecular analysis of blood meals from wild mosquitoes combined with avian census, and behavioural bioassays in laboratory. The latter methodology identifies intrinsic preferences, since it excludes potentially confounding variables such as environmental conditions, bird abundance and behaviour. Together, these findings suggest that blackbird and magpie (as preferred species), along with house sparrow and collared dove (as abundant species that are opportunistically fed upon)*,* have the potential to play a crucial role in the circulation and amplification of West Nile virus in Italy. In addition, blackbird represents a major host for other viruses transmitted by *Culex* mosquitoes which are closely related to West Nile virus, such as Usutu virus and Sindbis virus [55]. The importance of blackbird in northern Italy therefore, mirrors the importance of the American robin (*Turdus migratorius*) in the United States [3,14,17,18,22], and suggests that the true thrushes of the genus *Turdus* may play a key role in the transmission of zoonotic pathogens transmitted by *Culex* mosquitoes.

In Veneto, only a part of the blackbird breeding population present in agricultural and urban areas is resident. After breeding (March to July), many juveniles and adults move from nesting areas to sites rich in fruiting plants (e.g. *Sambucus nigra*, *Viburnum lantana*, *Cornus sanguinea*, *Prunus spinosa*, *P. padus*) where they moult and accumulate fat reserves prior to the autumnal migration [42,43]. Because of these movements, only a relatively small number are available as potential hosts for *Cx. pipiens* mosquitoes. On the other hand, house sparrow, magpie and collared dove are resident, but the density of their populations increases at the end of the summer because newly born juveniles add to the adult populations [44]. Although a preference for blackbird was consistent within the current study (among sites, seasons, and methods), the degree of preference for blackbird and for other species shifted both seasonally, and with habitat. The highly variable nature of mosquito feeding preference suggests that broader inferences about the significance of blackbird, or genus *Turdus* in general, must be made cautiously: similar studies in Europe should be carried out in other areas and habitats.

The behavioural bioassays confirmed the results obtained in the field for blackbird, suggesting that the high feeding preference index is the result of intrinsic mosquito preference. On the other hand, the behavioural bioassays did not confirm preferences for magpie in respect to other species, suggesting that the observed feeding preference index in the field strongly depends on host ecology/behaviour of this species in the area. However, since there is still a lack of knowledge of the chemical composition of body odours of many European bird species, it could be interesting in future to repeat the behavioural bioassay using synthetic volatiles identified from the headspace extracts of a larger number of local birds species. The chemical analysis of their headspace extract solutions and subsequent electrophysiological recordings could help in selecting the single volatile compounds involved in host recognition and in evaluating their activity even at longer range in either semi-field or field conditions [47].

Overall these results suggest that while mosquito feeding behaviour in the field can be partially ascribed to intrinsic feeding preferences, it is a plastic pattern which can be overridden by environmental circumstances such as avian abundance or behaviour [20]. For instance, the observed avoidance of the barn swallow and the common house martin can be explained by their behaviour: both are insect-eating birds that feed on the wing, and are largely inaccessible to feeding mosquitoes for a significant fraction of the day [56]. Differences in mosquito preference between blackbird and common starling, both of which feed on or near the ground, can perhaps be partly explained by the crepuscular foraging habits of the former, which fits with *Culex* mosquito feeding habits [16,57], and the diurnal feeding habits of the latter [44]. Recent studies carried out in North America demonstrated that *Culex* mosquitoes feed more actively on species roosting at high altitude (such as American robin) rather than at the lower altitude, so that variation in habitat use by host and vectors and social aggregation by hosts influence vector-host interaction [22].

A sharp decline in the availability of blackbirds late in summer during the mosquito activity season (i.e. July-September) was reflected by a decrease in blackbird blood meals. However, when abundance is taken into account it is apparent that the decrease in blood meals is less than would be expected, as revealed by an increased preference index. At the same time, we observed a sharp increase in feeding on magpies. Our analyses suggest that the overall apparent preference for magpies is entirely driven by the late season preference. The observed increase in magpie feeding preference is likely to be driven in part by the decreased availability in blackbirds, but also by the increase in communal roosting in late summer/early fall that follows the end of the magpie breeding season [58]. On the other hand, blackbirds maintain their home range throughout the year even if during winter some latitudinal migration weather dependent may occur. Clustering around winter food resources might occasionally occur but the species, in the study area, does not properly roost or nest in colonies [59]. This interpretation is in agreement with others [16] highlighting that, for nocturnal or crepuscular feeding vectors as *Cx. pipiens*, the over-utilization of a host species can arise from an overlap between mosquito microclimate selection and host roosting behaviour.

Human cases of WNV in Northern Italy tend to peak in August-September (see e.g. [60]). This seasonality may reflect the variation in feeding preference by mosquitoes, as observed in the USA where a rise in human WNV infections coincides with a shift in feeding behaviour following the dispersal of the American robin [17]. Further studies conducted in Alabama (southern USA) showed that host phenology and winter temperatures may also contribute to the temporal shift in mosquito feeding pattern [21].

Mosquito feeding indices reflect a stronger preference for blackbirds in rural areas, and for magpies in peridomestic environments. The preference for magpies in peridomestic areas, where the contact rate with humans is higher, suggests that they may be an important bridge-host for WNV transmission to humans. Data from the WNV surveillance program carried out on sinantropic corvids in the WNV circulation area of northern Italy has evidenced that magpies contribute 70% of the WNV positivity in corvids, suggesting a significant role for magpies in WNV transmission [61]. In addition, the potential importance of magpies in WNV amplification and transmission could be supported by our observations that the magpie feeding index is significantly greater in areas with known WNV circulation, compared to sites where WNV has never been detected. However, this hypothesis needs to be validated. Since WNV positive and negative sites in the study area were spatially clustered, alternative hypotheses (such as different patterns in avian community, habitat, and climatic conditions) may explain the observed differences in WNV circulation.

There is a considerable knowledge gap in Europe in relation to the reservoir competence for WNV and this limits the possibility to further model the risk for WNV transmission in relation to the local host community composition and abundance. Blackbirds and magpies have been found infected or at least exposed (seroconversion) to WNV in Europe on several occasions [62-66]. However, studies on their competence for the strain of WNV circulating in Europe are still very limited. Experimental studies on house sparrow both in USA and in Europe showed that this species may develop high levels of viraemia. However, competence may differ depending on the virus strain tested, and host competence can vary geographically [67,68]. Species belonging to genera *Turdus* and *Pica* are highly competent hosts for the WNV strain circulating in North America [68,69], but unfortunately no studies have been performed on these species so far with European WNV strains. For these reasons, estimates of host competence obtained in different epidemiological contexts must be treated with caution.

Despite the acknowledged limitations, we believe that the current study provides new and valuable insights into the ecology of *Cx. pipiens.* Given the key role of *Cx. pipiens* as the main vector of WNV and other emerging flaviviruses such as Usutu virus, these findings are crucial in order to implement targeted eco-epidemiological research and surveillance.

## Conclusions

West Nile virus is spreading in Europe and although the number of human cases is still sporadic, it is fundamental to understand the ecological mechanism driving its emergence and spread, including the contribution of different avian species as feeding hosts of *Cx. pipiens* mosquitoes in order to identify potential virus amplifiers. Here, we found that the blackbird (*Turdus merula*) is the most preferred species by *Cx. pipiens* both in the field and laboratory experiments. However, later in the mosquitoes activity season (from July to October), the abundance of blackbird drops significantly and *Cx. pipiens* preferences shift toward the Eurasian magpie (*Pica pica*). Magpie is highly preferred by *Cx. pipiens* in sites closer to human settlements indicating that this species may contribute to WNV seasonal spill-over events to human and domestic animal species.
